# Graphlet Based Metrics for the Comparison of Gene Regulatory Networks

**DOI:** 10.1371/journal.pone.0163497

**Published:** 2016-10-03

**Authors:** Alberto J. M. Martin, Calixto Dominguez, Sebastián Contreras-Riquelme, David S. Holmes, Tomas Perez-Acle

**Affiliations:** 1 Computational Biology Lab, Fundación Ciencia & Vida, Santiago, Chile; 2 Centro Interdisciplinario de Neurociencia de Valparaíso, Universidad de Valparaíso, Chile; 3 Center for Bioinformatics and Genome Biology, Fundación Ciencia & Vida and Facultad de Ciencias Biologicas, Universidad Andres Bello, Santiago, Chile; Universite Paris-Sud, FRANCE

## Abstract

Understanding the control of gene expression remains one of the main challenges in the post-genomic era. Accordingly, a plethora of methods exists to identify variations in gene expression levels. These variations underlay almost all relevant biological phenomena, including disease and adaptation to environmental conditions. However, computational tools to identify how regulation changes are scarce. Regulation of gene expression is usually depicted in the form of a gene regulatory network (GRN). Structural changes in a GRN over time and conditions represent variations in the regulation of gene expression. Like other biological networks, GRNs are composed of basic building blocks called *graphlets*. As a consequence, two new metrics based on graphlets are proposed in this work: REConstruction Rate (REC) and REC Graphlet Degree (RGD). REC determines the rate of graphlet similarity between different states of a network and RGD identifies the subset of nodes with the highest topological variation. In other words, RGD discerns how th GRN was rewired. REC and RGD were used to compare the local structure of nodes in condition-specific GRNs obtained from gene expression data of *Escherichia coli*, forming biofilms and cultured in suspension. According to our results, most of the network local structure remains unaltered in the two compared conditions. Nevertheless, changes reported by RGD necessarily imply that a different cohort of regulators (i.e. transcription factors (TFs)) appear on the scene, shedding light on how the regulation of gene expression occurs when *E. coli* transits from suspension to biofilm. Consequently, we propose that both metrics REC and RGD should be adopted as a quantitative approach to conduct differential analyses of GRNs. A tool that implements both metrics is available as an on-line web server (http://dlab.cl/loto).

## Introduction

Networks are everywhere [1]. They are used to represent complex data associations from different domains ranging from social interactions and technological developments up to biological systems [2]. In biological sciences, network representations are predominantly adopted to depict metabolic pathways [3], cell signaling cascades [4, 5], protein-protein interactions [6], and GRNs [7, 8]. GRNs are directed networks where nodes represent genes, and edges between nodes are present if a transcription factor (TF) encoded by a *source* gene regulates the expression of a *target* gene. Major applications of GRNs are intended to perform differential studies between realizations of biological systems over time and/or conditions. This approach has been successfully applied in several studies including comparisons between diseases and normal samples [9, 10], knockout and/or mutant models versus wild type [11], and comparisons between different developmental stages [12, 13]. Interestingly, classic approaches focus on the characterization of gene expression levels [14–16], disregarding changes in gene regulation and therefore, on the network structure.

Networks are composed of small building blocks called *graphlets*. Statistically over-represented graphlets are commonly called *motifs* [17]. Even though over-representation depends on the null model employed as baseline [18, 19], graphlets still represent local structural patterns that may be assigned functional roles [20]. Moreover, the existence of several graphlets has been functionally characterized in GRNs ranging from bacteria to higher animals [21–27]. Therefore, graphlets-based metrics could be used to describe and compare structural properties of biological networks [28, 29]. In consequence, previous work has proposed graphlet distribution [19, 30], graphlet degree distribution [28, 31, 32] and graphlet correlation distance [33]. Despite particularities, all these metrics reflect global properties of the network structure, disregarding local structural differences that may be useful to identify changes in the regulation of gene expression. To do so, the identification of subnetworks is a logical step forward. There are several methods to identify subnetworks that change in different non-directed network states, either based on graphlets [34] or based on other similarity metrics [35]. While the former methods focus on identifying graphlets that change in co-expression networks the latter rely on using the Hamming distance to identify changes between different co-methylation networks. On the other hand, node-based comparisons such as node degree [10] and node centralities [1, 36] can identify those nodes whose relationship with other components of the network changes in different states of the network. Furthermore, other approaches implicitly consider the dynamic properties of GRNs. In these dynamic methods, individual edges [37], or graphlets [38], are associated with a vector of temporal events that denotes their presence and absence over time.

Considering the relevance of graphlets, we have developed a graphlet based metric called REC that quantifies the rate of graphlets reconstruction to compare the local structure of GRNs. REC was also expanded to create the RGD, a metric that identifies the subset of nodes with the highest topological variation between different states of a network. This work describes the application of both REC and RGD to perform comparisons of condition specific GRNs that represent the growth of *E. coli* in two different conditions; in suspension and in biofilms. In doing so, the regulatory core of TFs and its neighborhood of target genes with the higher variation over time and conditions were identified, shedding light on the adaptation process occurring when *E. coli* transits from suspension to biofilm, and vice versa. Our method is freely available to the academic community as an on-line web server at http://dlab.cl/loto.

## Materials and Methods

### Expanding the definition of graphlets

**Fig 1:**
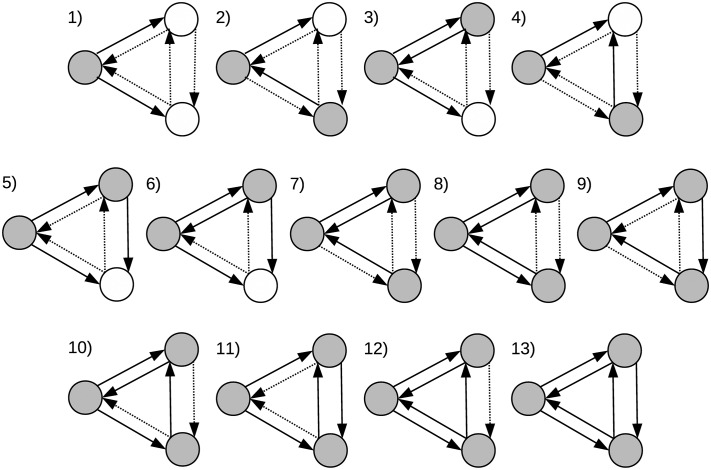
Graphlets composed by three nodes. The direction of edges indicate the direction of the transcription regulation. Straight black edges denote true interactions, and dashed edges depict false ones. Grey nodes represent genes that are required to be TF encoding genes, white nodes represent genes that do not require to code for TF. Adapted from [17].

In this study, graphlets are defined as small induced subgraphs formed by three nodes with at least two regulatory relationships (true edges) among them. Thus, 13 graphlets could be formed considering all possible connectivity patterns that meet the previous definition (Fig 1). Importantly, the classical definition of graphlets proposed in [17] was expanded by making equally relevant both the presence and absence of regulatory interactions between nodes. Under this definition, all graphlets depicted in Fig 1, except number 13, require non-existing regulatory relationships (false edges) between nodes. Another important characteristic of these graphlets is the number of nodes that necessarily must encode for TFs. While type 1 is the only graphlet that requires a single gene encoding for a TF, types 2 to 6 require at least two genes, and types 7 to 13 require three genes to encode for TFs.

Of note, our definition of graphlets could be further extended to consider graphlets formed by more than three nodes. However, connected graphlets formed by more than three nodes do include at least a graphlet formed by three nodes [39]. Thus, three nodes graphlets are implicitly included in larger graphlets. Moreover, considering graphlets with more than three nodes is an np-hard problem that requires non polynomial time as a function of the extra nodes used to define larger graphlets.

### Comparing the structure of GRNs

To compare different states of GRNs, one needs a formal framework. Let *G* be a state of a GRN with *V* nodes and *E* edges, we want to compare its local structure (topology) with another state of the network *G*′. In this way, *G*′ should be composed of the same set of nodes *V*, or a subset of them *V* ∣ *V*′ ⊆ *V*, differing from *G* in the set of edges, *E*′. Thus, a comparison between the local topology of *G* = (*V*, *E*) and *G*′ = (V*, *E*′) should be performed, where V* represents either *V*′ or *V*. To do so, we propose a novel metric called REC. REC accounts for the rate of graphlets reconstruction (see below): it first enumerates the number of true and false edges between the same triplets of nodes in both states, then it compares them and summarize the comparison into a single value. Therefore, REC provides a new approach to determine variations in the local structure of different states of the network. These states represent a biological system in different conditions, either temporal or environmental. Further elaboration of REC, led to the development of RGD, the average REC for all graphlets in which the same node participates. RGD can be used to identify the subset of nodes with the highest topological variation between different states of a network, highlighting how the GRN was rewired.

#### Accessing the rate of graphlets reconstruction

The rate of graphlets reconstruction (REC) between network states *G* and *G*′ measures how similar is the connectivity pattern between edges spanning through the same triplet of nodes in both states. Consistently with binary classification problems, existing edges (true) and absent ones (false) are transformed into numerical values, 1 and 0, respectively. Let *A* and *B* be the adjacency matrices representing the induced subgraph formed by the same triplet of nodes in *G* and *G*′, and *a*_*ij*_ and *b*_*ij*_ equivalent elements of *A* and *B*, REC for a single graphlet is calculated using Eq (1). Considering our definition of graphlets, *N* = 3 and *N* × (*N* − 1) = 6 in Eq (1). In other words, REC is equivalent to the percentage of correctly classified edges, or accuracy, belonging to the subgraphs formed by the same three nodes between the two compared network states. Thus, REC ranges from 0 to 1; 0 indicating total disagreement and 1, a perfect match.

$$\begin{array}{c} REC=1-\frac{1}{N(N-1)}\sum_{i,j=1,i\neq j}^{N}\left|a_{ij}-b_{ij}\right|; \end{array}$$ (1)

REC compares edges, so for those cases in which a node is only present in one of the network states and absent in the other one, all interactions of this node in the state where it is absent are considered as false edges.

#### Identifying network rewiring

Importantly, REC can be averaged over all graphlets in which a node participates, *K*, by calculating the RGD as Eq (2)

$$\begin{array}{c} RGD=\frac{1}{K}\sum_{1}^{K}(1-\frac{1}{N(N-1)}\sum_{i,j=1,i\neq j}^{N}\left|a_{ij}-b_{ij}\right|); \end{array}$$ (2)

RGD can be applied to identify those nodes that exhibit the largest variation in their local connectivity and their neighborhood of genes. Therefore, RGD also gives a way to determine the subnetworks whose expression and regulation should be affected by the changes driving the adaptation to the conditions depicted in the compared network states.

#### Graphlet assignment

Since graphlets involve three nodes, a brute force implementation would have a complexity of *O*(*n*^3^), where *n* is the total number of nodes in the network. In GRNs, edges only connect genes encoding TFs to their targets, therefore it is possible to reduce the complexity to find graphlets to *O*(*t* ∗ *n*^2^), where *t* is the number of genes encoding TFs. In our implementation, networks are represented using an adjacency list. The adjacency list contains only true edges arising from genes encoding TFs, thus, using less memory resources and allowing to take advantage of GRNs being sparse. Self-connections are not included in the adjacency list, so the three nodes forming a graphlet are forced to represent different genes. For each gene encoding TFs, a loop over each of its true connections, stored in the adjacency list, is carried out. This reduces the computational cost in finding the first true edge of each graphlet from *O*(*t* ∗ *n*) to *O*(*t* ∗ *k*), where *k*, the number outgoing true connections or outdegree of each gene encoding TFs, is < < *n* for most of the nodes and at most equal to *n*. Therefore, the total estimation of computational complexity of the algorithm to find graphlets becomes *O*(*t* ∗ *k* ∗ *n*).

Further details about how REC and RGD are calculated, including an example of how to use both metrics to compare two small networks, are shown in S1 Fig. Basal values of REC and RGD on randomized networks and a table resuming the efficiency and averaged running times on random GRNs of different sizes are shown in S1 Text.

### Generating Condition specific GRNs

Condition specific GRNs were built based on gene expression experiments following a similar approach to that adopted in [40]. To this end, edges found in a gold standard or reference network were kept only when they originate from nodes encoding for TFs whose expression is corroborated by an experimental procedure. The GRNs analyzed were built by using data from time series experiments generated to study differential gene expression between *E. coli* forming biofilms and cultured in suspension (GEO accession GDS2768, [41]). In this study, gene expression was evaluated in the two aforementioned conditions at 4, 7, 15 and 24 hours. To determine whether genes encoding TF are expressed or not, a threshold of 0.05 for the P-value of the probeset signal (as reported in the expression measurements) was employed. When there is no match between the gene names in the experiment and the reference network, the names in the latter were kept.

The gold standard for the *E. coli* GRN was constructed from RegulonDB [42] version 8.7. Data from this database was used to determine all true edges known for the entire *E. coli* genome as follows. First, the list of TFs was linked to their product IDs and the product IDs to their respective genes. This step determined which genes encode for TFs. In the second step, each gene encoding TFs was linked to their target genes; those genes whose expression is regulated by the TF. TF IDs were linked to their regulons, regulons to their functions, functions to promoters, promoters to transcription units and, finally, each transcription unit to its gene IDs. All genes encoding TFs and all genes not encoding TFs with at least one true connection were kept. Other gene products that also regulate gene expression, such as sRNAs, were not taken into account for the sake of simplicity, but they could be easily included when GRNs are created. RegulonDB only contains information about true edges (actual regulatory interactions); therefore, false edges were assumed to occur between all nodes not linked by a true edge. An image of this gold standard network is shown in S2 Fig.

All the condition specific and gold standard GRNs can be downloaded from the website at http://dlab.cl/loto.

### Network visualization and centrality measures

Networks images were created using Cytoscape [43]. Centrality metrics commonly applied to describe nodes in a network were also computed in Cytoscape by using its built-in NetworkAnalyzer [36]. The metrics compared with RGD were average shortest path length, betweenness centrality, closeness centrality, clustering coefficient, eccentricity, edge count, in-degree, out-degree, stress centrality and neighborhood connectivity described in [1, 36] and defined in S3 Table). To do so, they were calculated for each node in both networks to then compare their values, i.e., calculating the absolute value of the difference. This procedure was followed for each network comparison, so the correlation of each metric with RGD was calculated.

### Statistical tests

The R package [44] (version 3.0.2) was used to perform statistical tests and to compute correlation coefficients. A one-way ANOVA test was applied to evaluate the statistical significance of the rate of RGD variation, with a P-value of 0.05 considered as threshold for significant variations. The correlation coefficients employed to establish the existence of a relationship between RGD and differences in the centrality metrics mentioned above were Spearman’s rank correlation (*ρ*), Kendall’s rank correlation (*τ*) and Pearson correlation (r).

## Results and Discussion

### Assessing the dynamic behavior of GRNs

The occurrence of graphlets in the condition specific networks (see Methods) was calculated to reveal how the local structure of GRNs changes over time and in different conditions. The number of nodes, true edges and TFs, forming each state of the condition specific networks and the gold standard, are described in S1 Table. Interestingly, the number of genes encoding TFs in the case of biofilm was always higher than that of suspension, with the only exception of the networks at four hours. Consequently, this trend was also observed for the number of nodes and true edges forming the networks.

The occurrence of each type of graphlets and the number of genes not forming any graphlet (NOG) in each condition specific GRN is shown in S2 Table. It is worth noting that the fraction of nodes that participate in any graphlet remained almost invariable in all the networks (at most 0.5% of the genes do not form graphlets), demonstrating that graphlet-based metrics do actually consider most of the nodes in GRNs. Notably, all graphlet types but type 9, occurred in these condition specific GRNs. The absence of type 9 graphlets was expected as they were also absent in the gold standard. In addition, the occurrence of each graphlet type maintained approximately the same ratio as in the gold standard, but exhibited a reduction proportional to the also diminished number of target nodes and genes encoding TFs. Three graphlets of type 12 and one graphlet of type 13 found in the *E. coli* gold standard were also detected in the condition specific networks. This indicates that at least the portions of the GRN formed by these four graphlets remained unaltered independently of the time point and condition studied.

The average REC per graphlet type was also calculated by considering equivalent time points, as shown in S3 Fig. The relatively small variation of the average REC indicates that the majority of the local topology remains unaltered over time. As observed in Fig B in S3 Fig, it seems that, on average, most of the graphlets present in the suspension networks were also maintained in biofilm. The largest difference observed is in both cases at 15 hours, with less pronounced changes at 7 and 24 hours. Interestingly, most of the variation observed affects graphlets that require their three forming nodes to encode for TFs (types 8, 10 and 11).

**Fig 2:**
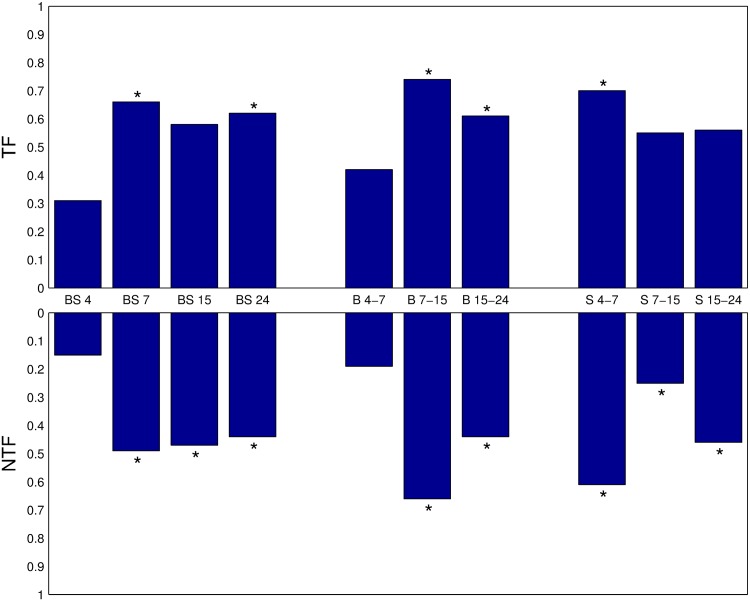
Statistical significance of RGD variation. Fraction of genes with RGD < 1.0 for genes participating in at least one graphlet comparing different conditions and time points. BS: biofilm versus suspension, for all four time points analyzed. B: biofilm, at consecutive time points. S: suspension, at consecutive time points. TF: genes coding for TFs. NTF: genes non coding for TFs. Statistically significant P-values obtained from a one way ANOVA test (0.05 threshold) are marked with an ∗ .

The proportion of genes showing changes in their local topology according to their RGD was determined (Fig 2) to study in more depth changes occurring in the structure of the GRNs. All genes were considered for further analysis. A one-way ANOVA statistical test was performed in order to obtain a P-value to establish the significance of the comparisons. Notably, the number of genes encoding TFs exhibits a higher variation than that of the genes not coding for TFs. When considering the statistical analysis for genes coding for TFs, significant variations occurred at 7 and 24 hours, with 66% and 62% variation, respectively. These results support the notion that the adaptation process between both conditions requires dramatic changes in the topology of the regulatory core of the GRN [45–47]. Interestingly, these results appeared to disagree with the original analyses [41], in which the largest difference on gene expression levels was reported at 4 and 7 hours, with 3.2% and 2.5% of the genes changing their expression, respectively. Despite this apparent difference, it should be noted that instead of changes on gene expression, RGD reports changes of the network structure. These changes imply that a different cohort of regulators (i.e. TFs) appear over time suggesting that the GRN had been rewired.

Genes not coding for TFs showed a different behavior: the largest number of genes with a RGD < 1.0 occurs at 7 hours (49% of the genes), followed by 15 hours (47%), then at 24 (44%) and the smallest at 4 hours (15%). As evident, compared to the rate of variation of genes coding for TFs, the adaptation between both conditions requires a smaller change to the local structure of genes not coding for TFs in the GRN.

In a comparison between consecutive time points, the largest variation in RGD for genes coding for TFs occurred between 7 and 15 hours (74%), and between 4 and 7 hours (70%), for biofilm and suspension respectively. In the case of genes not coding for TFs, the largest variation in RGD occurred between 7 and 15 hours in biofilm (66%) and between 4 and 7 for suspension (61%). These results indicate that the majority of the changes on the structure of the GRN occurs during the transition from early stages of development to mature biofilms (7 to 15 hours) and earlier when *E. coli* was cultured in suspension (4 to 7 hours).

### Comparison of RGD with other centrality metrics

To determine whether a relationship between RGD and the variation of node centralities exists or not, we studied the comparisons of condition specific networks at 15 hours. Notably, REC calculated at this point exhibited the largest variation and therefore, the local structure of GRNs should display the largest topological variations between the two analyzed conditions (see S3 Fig). Three different correlation coefficients were employed: Kendall and Spearman correlations that compare order relationships and Pearson correlation that measures the strength of the linear relationship.

**Fig 3:**
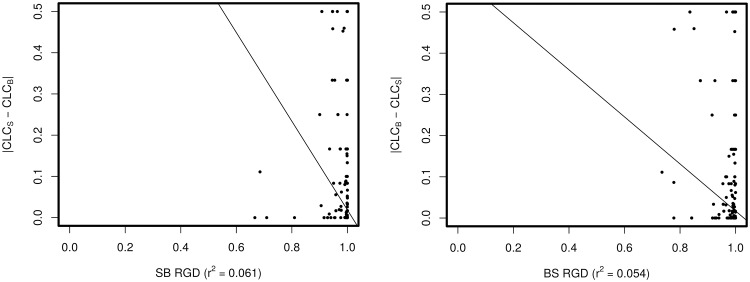
RGD versus absolute value of the change in CLustering Coefficient (CLC) at 15 hours. Left panel shows values of the metrics calculated using the suspension network as reference and right panel using the biofilm condition as reference in the comparison. Regression line calculated with R using as dependent variable RGD, regression coefficients between brackets.

Absolute values of the correlation coefficients between RGD and the centralities were in the [0.15, 0.65], [0.13,0.6] and [0.06,0.45] ranges for Spearman, Kendall, and Pearson correlations respectively (see S3 Table). These values indicate the existence of a weak relationship between several of these node centralities and RGD. However, the strength of this relationship varies depending on the metric and network used as reference in the comparison. Interestingly, the results of the correlation analysis also indicated the existence of discordance between RGD and the variation in centrality measures. This partial disagreement was confirmed by looking at the number of nodes identified simultaneously by RGD and by node centrality metrics and the number of nodes only identified by either RGD or by the centralities. As shown in S3 Table, with the only exception of neighborhood connectivity, the number of nodes identified by the centralities is smaller than the number of nodes identified by RGD. In other words, RGD is capable to identify more topological changes than any of the assayed centrality measures. Centrality metrics can be separated into two groups, those that require the calculation of shortest paths and those that are not based on shortest paths. Metrics based on shortest paths—average shortest path length, betweenness, closeness, eccentricity and stress centrality—consider the relationship of each node with all other nodes in the same connected component of the network. On the other hand, degrees, clustering coefficient and neighborhood connectivity only take into account the local topology of each node. Since our graphlet-based metrics reflect the local topology of a node, it is not surprising that the metrics that do not rely on shortest paths show better correlation and higher overall agreement with RGD. Centrality values were also plotted against the respective RGD values for each gene (shown after S3 Table in the same file). These comparisons were performed to investigate if the correlation coefficients shown in S3 Table were dominated by any trend in the values of the metrics. As an example, Fig 3 shows the relationship of clustering coefficient with RGD on the same comparisons used to build S3 Table. As seen, in a similar way with the other metrics (see the figures below S3 Table), the relationship between RGD and clustering coefficient does not follow any obvious trend, thus reinforcing the evidence to demonstrate that our graphlet-based metrics and node centralities are in fact different approaches.

### Using RGD to identify nodes encoding TFs with the highest variation in their local topology

**Fig 4:**
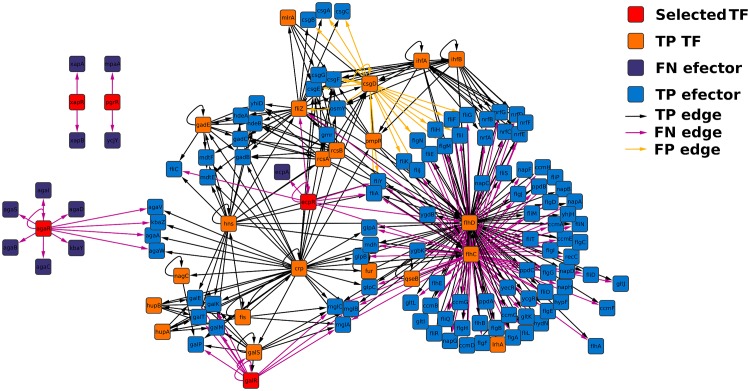
Merged sub-network of the TFs with the lowest RGD using biofilm network at 15 hours as reference. The TF encoding genes identified using their RGD are colored in red; other TFs are colored in orange and they are named True Positive (TP) because their expression was detected in the two compared networks; effector genes, those that do not code TFs are colored in purple if their expression was detected only in the reference network (False Negative (FN) nodes) and in blue if their expression was detected in both networks; with respect to the edges, they are colored in black if they were present in both networks (TP edges), light purple if they were found only in the reference network (FN edges) and yellow when they were detected only in the compared network (False Positive (FP) edges). The same image but with TP edges removed is shown in S4 Fig.

**Fig 5:**
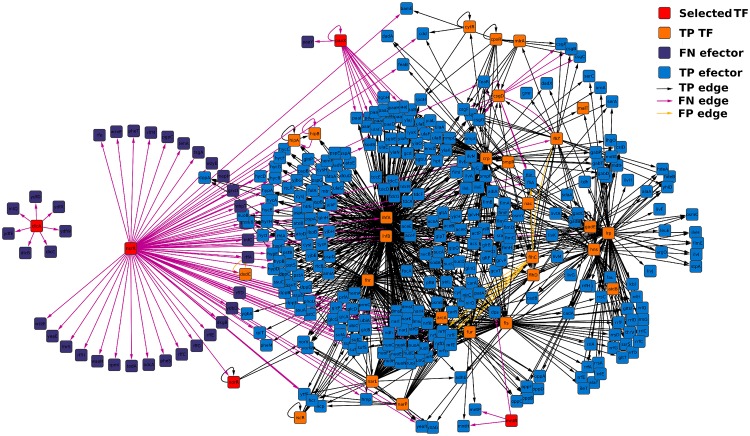
Merged sub-network of the TFs with the lowest RGD using suspension network at 15 hours as reference. The TF encoding genes identified using their RGD are colored in red; other TFs are colored in orange and they are named TP because their expression was detected in the two compared networks; effector genes, those that do not code TFs are colored in purple if their expression was detected only in the reference network (FN nodes) and in blue if their expression was detected in both networks; with respect to the edges, they are colored in black if they were present in both networks (TP edges), light purple if they were found only in the reference network (FN edges) and yellow when they were detected only in the compared network (FP edges). The same image but with TP edges removed is shown in S5 Fig.

The five genes encoding for TFs with the largest local topology variation according to their RGD using biofilm and suspension as references are shown in Table 1. This table shows both the RGD of each gene and their graphlet degree, i.e., the number of graphlets in which each of these genes participate. The subnetworks composed of those genes found in the same graphlets as the genes encoding TFs with the lowest RGD using biofilm as reference, are shown in Fig 4. On the other hand, Fig 5 shows the subnetworks belonging to those genes encoding TFs with the lowest RGD using suspension as reference. There are 26 genes encoding TFs in the largest connected component of the subnetwork identified using biofilm as reference. The subnetwork identified using suspension as reference contains 31 genes encoding TFs in its largest component. Thirteen genes coding for TFs appear in the two subnetworks, indicating that RGD was able to identify the subnetworks that better explain the differences between the two compared conditions independently of which condition was employed as reference. Since both largest connected components share as many nodes encoding for TFs, it is suggested that RGD was able to identify the subnetworks that better explain the differences between the two compared conditions independently of which condition was employed as reference. The majority of the genes encoding TFs with the lowest RGD were interconnected in this large component. This implies both an interdependency of their regulation and also an exquisite fine-tuning promoted by a small number of TFs. Noticeably, the only genes coding for TFs whose subnetworks were disconnected from the largest connected component were already disconnected in the gold standard. These genes were *pgrR* and *xapR* in the case of biofilm as reference, whereas for suspension as reference, the only disconnected gene coding for TFs is *dicA*. To further explore the role of these genes, their regulation, co-regulation and interaction with other genes forming the subnetworks, their functions were reviewed using EcoCyc [48] and RegulonDB [42], as described in S2 Text. Interestingly, most of the genes coding for TFs identified by RGD, were found to actually regulate the expression of genes involved in several of the main functional clusters mentioned in the original article [41]. These clusters were motility and *fimbriae* (*ecpR*); stress response genes (*pgrR*, *mntR*, *nsrR* and *norR*); transport genes (*galR*, *agaR*, *xapR*, *nsrR* and *paaX*); extracellular matrix (*pgrR*); respiratory genes (*nsrR*); and indole and sulfur genes (*nsrR*). Notably, the explicit employment of graphlets to uncover these genes encoding TFs allowed, not only the identification of their target genes, but also to establish the relationship with their regulators. Thus, RGD identified key TFs that become predominant when *E. coli* transits between biofilm and suspension, shedding light on how the GRN changes its topological structure to allow the adaptation to new conditions.

**Table 1:** Genes coding for TFs with the lowest RGD comparing the two networks at 15 hours.

Biofilm as reference			Suspension as reference		
TF	Graphlet Degree	RGD	TF	Graphlet Degree	RGD
*pgrR*	1	0.667	*dicA*	21	0.667
*xapR*	1	0.667	*nsrR*	3662	0.686
*agaR*	49	0.680	*norR*	93	0.710
*galR*	66	0.735	*paaX*	114	0.719
*ecpR*	216	0.755	*mntR*	9	0.778

## Conclusion

As a whole, our results indicate that both REC and RGD are good quantitative metrics that can be used to determine the topological similarity between GRNs representing the same system under diverse conditions. Notably, RGD was able to identify the nodes and the subnetworks that undergo the largest topological variations when *E. coli* transits between biofilm and suspension. Changes on these networks account for the functional variations that are involved in phenotypic adaptations to the aforementioned conditions. Thus, our method proved its usefulness by providing a complementary approach to methodologies based on quantifications of alteration in gene expression levels or centrality metrics. Of note, using RGD we discovered a large subnetwork connecting TFs with the lowest RGD. The existence of this connected component implies both an interdependency of the regulation of the genes that are part of it and also an exquisite fine-tuning promoted by a small number of TFs. These observations open up new opportunities for experimental investigation of the regulation of gene expression.

## Supporting Information

S1 Fig Topological comparison of two small networks: Example on how REC and RGD are used to compare two simple networks. A) shows the two small networks that are been compared; B) identification of each graphlet in each network; C) calculation of REC for each graphlet; D) computation of how RGD for node B. Black edges denote true interactions, and red-dashed edges depict false ones.
(PDF)

S2 Fig RegulonDB 8.7 *E. coli* gold standard. This image shows visually the gold standard used through the study with different purposes. Transcription Factor encoding genes are colored in red. The eleven nodes that do not form graphlets are shown within a red box.
(PDF)

S3 Fig Comparison of equivalent time points under the two conditions studied using REC for each graphlet type. The plots in the first row show REC cumulative results for each graphlet type using the four states of the condition specific networks for the biofilm formation as reference network (sub-figure A) or suspension (sub-figure B).
(PDF)

S4 Fig Merged sub-network of the TFs whose RGD varies the most using biofilm network at 15 hours as reference. Only FP and FN edges are shown. Color codes are the same as in Figs 4 and 5 in the main text.
(PDF)

S5 Fig Merged sub-network of the TFs whose RGD varies the most using suspension network at 15 hours as reference. Only FP and FN edges are shown. Color codes are the same as in Figs 4 and 5 in the main text.
(PDF)

S1 Table Components of the condition specific and gold standard GRNs. This table shows the number of TFs, nodes (genes) and true edges occurring at four time points of the GRN of *E. coli* cultured in suspension and forming biofilms.
(PDF)

S2 Table Graphlets in condition specific and gold standard GRNs. This table shows the occurrence of each graphlet type and the number of genes that do not form any graphlet (NOG) along four time points when *E. coli* was cultured in suspension, forming biofilms and in the entire gold standard. Graphlets of types 9 are absent in all these condition specific networks.
(PDF)

S3 Table Comparison of RGD with other centrality metrics in condition specific networks at 15 hours. Columns in this table show, from left to right, Spearman’s correlation (*ρ*); Kendall’s correlation (*τ*); Pearson correlation (r); The rate of disagreement (d = nodes identified only by one metric/# nodes); The rate of disagreement for nodes with variations in the metric (dc = nodes identified only by one metric/nodes identified only by one or the two metrics metric); The number of nodes identified by both metrics (Both); only by RGD (RGD); only by the other metric (M); and the total number of nodes (#). The comparisons were performed using only those nodes for which both RGD and the centrality measurements could be computed using the Biofilm networks as reference (BS) and the Suspension network as reference (SB). Centrality metrics are (Cent): Average Shortest Path Length (ASPL); Betweenness Centrality (BC); Closeness Centrality (CC); Clustering Coefficient (CLC); Eccentricity (E); Edge Count (EC); Indegree (ID); Outdegree (OD); Neighborhood Connectivity (NC); and Stress Centrality (SC). See below for a definition of the centralities used. This file also contains the definition of the centrality metrics employed and several figures showing the comparison of these centrality metrics versus RGD on the comparisons performed using the biofilm and suspension networks at 15 hours as reference.
(PDF)

S1 Text Assessing REC and RGD on comparisons of random networks. This text explains the procedure followed to generate random GRNs of any given size and to randomize the *E. coli* gold standard. The file also shows the performance of the method with respect to network size and the values of REC and RGD on comparisons of randomized *E. coli* with the reference network.
(PDF)

S2 Text Functional characterization of genes coding TFs with lowest RGD at 15 hours. (PDF)
