# Supplementary material for: Graphlet Based Metrics for the Comparison of Gene Regulatory Networks
Source: PLoS One. 2016 Oct 3;11(10):e0163497. doi: 10.1371/journal.pone.0163497 (PMC5047442; doi:10.1371/journal.pone.0163497)
Supplement: S1 Fig — A) shows the two small networks that are been compared; B) identification of each graphlet in each network; C) calculation of REC for each graphlet; D) computation of how RGD for node B. Black edges denote true interactions, and red-dashed edges depict false ones. (PDF) [file pone.0163497.s001.pdf]

Graphlet Based Metrics for the Comparison of Gene Regulatory Networks: Fig S1: Topological comparison of two small networks

Alberto J.M. Martin, Calixto Dominguez, Sebastián Contreras-Riquelme, David S. Holmes and Tomas Perez-Acle

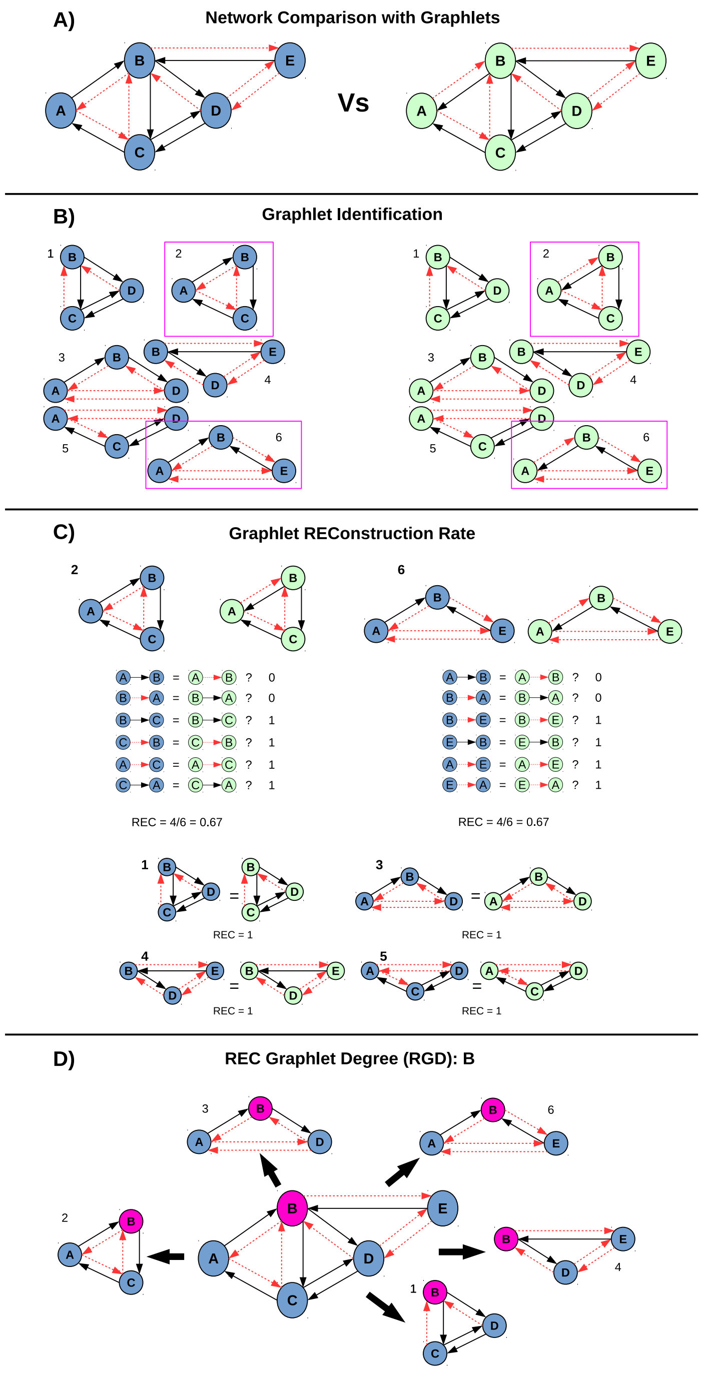

$$RGD_B = (REC_1+REC_2+REC_3+REC_4+REC_6)/5 = (1+0.67+1+1+0.67)/5 = 0.868$$

Node	# Graphlet	RGD
A	4	0.835
B	5	0.868
C	3	0.89
D	4	1
E	2	0.835

Example on how REC and RGD are used to compare two simple networks. A) shows the two small networks that are been compared; B) identification of each graphlet in each network; C) calculation of REC for each graphlet; D) computation of how RGD for node B. Black edges denote true interactions, and red-dashed edges depict false ones.
