# Supplementary material for: Graphlet Based Metrics for the Comparison of Gene Regulatory Networks
Source: PLoS One. 2016 Oct 3;11(10):e0163497. doi: 10.1371/journal.pone.0163497 (PMC5047442; doi:10.1371/journal.pone.0163497)
Supplement: S2 Fig — This image shows visually the gold standard used through the study with different purposes. Transcription Factor encoding genes are colored in red. The eleven nodes that do not form graphlets are shown within a red box. (PDF) [file pone.0163497.s002.pdf]

Graphlet Based Metrics for the Comparison of Gene Regulatory Networks:
Fig S2: RegulonDB 8.7 gold standard

Alberto J.M. Martin, Calixto Dominguez, Sebastián Contreras-Riquelme, David S. Holmes and Tomas Perez-Acle

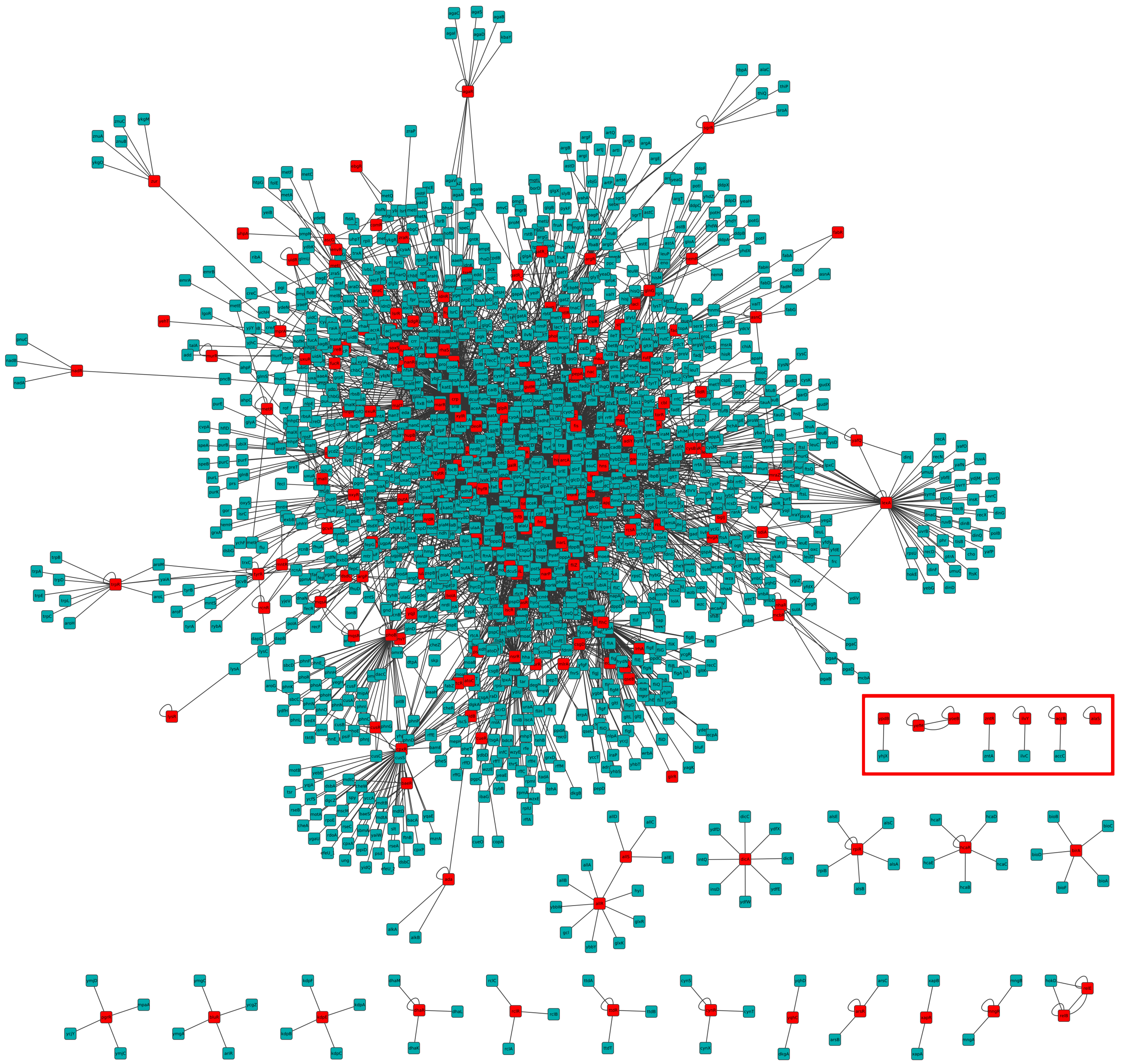

E. coli **gold standard network.** This image shows visually the gold standard used through the study with different purposes. Transcription Factor encoding genes are colored in red. The eleven nodes that do not form graphlets are shown within a red box.
