# Supplementary material for: Graphlet Based Metrics for the Comparison of Gene Regulatory Networks
Source: PLoS One. 2016 Oct 3;11(10):e0163497. doi: 10.1371/journal.pone.0163497 (PMC5047442; doi:10.1371/journal.pone.0163497)
Supplement: S3 Fig — The plots in the first row show REC cumulative results for each graphlet type using the four states of the condition specific networks for the biofilm formation as reference network (sub-figure A) or suspension (sub-figure B). (PDF) [file pone.0163497.s003.pdf]

Graphlet Based Metrics for the Comparison of Gene Regulatory Networks:
Fig S3: Comparison of equivalent time points under the two conditions studied using REC for each graphlet type

Alberto J.M. Martin, Calixto Dominguez, Sebastián Contreras-Riquelme, David S. Holmes and Tomas Perez-Acle

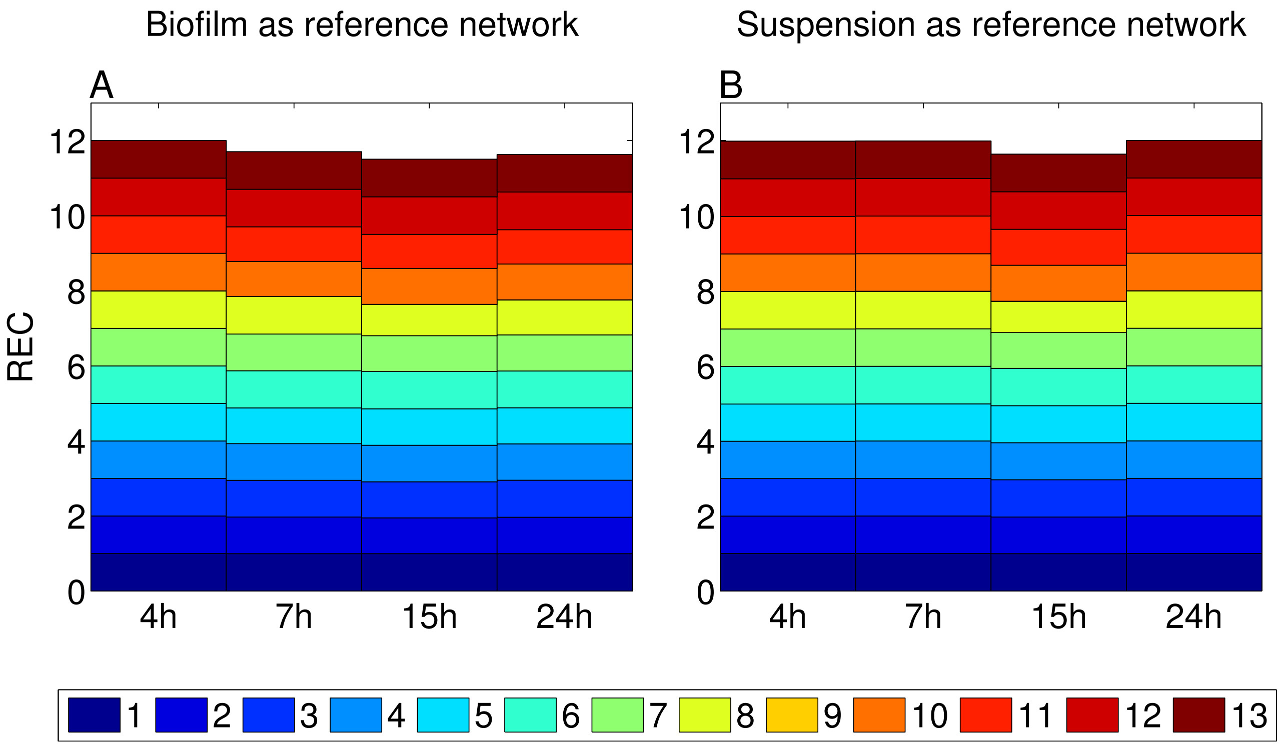

Comparison of equivalent time points under the two conditions studied: REC. The plots in the first row show REC cumulative results for each graphlet type using the four states of the condition specific networks for the biofilm formation as reference network (sub-figure A) or suspension (sub-figure B).
