# Supplementary material for: Graphlet Based Metrics for the Comparison of Gene Regulatory Networks
Source: PLoS One. 2016 Oct 3;11(10):e0163497. doi: 10.1371/journal.pone.0163497 (PMC5047442; doi:10.1371/journal.pone.0163497)
Supplement: S4 Fig — Only FP and FN edges are shown. Color codes are the same as in Figs 4 and 5 in the main text. (PDF) [file pone.0163497.s004.pdf]

# Graphlet Based Metrics for the Comparison of Gene Regulatory Networks: Fig S4: Merged sub-network of the Transcription Factors (TFs) whose REC graphlet degree (RGD) varies the most using biofilm network at 15 hours as reference.

Alberto J.M. Martin, Calixto Dominguez, Sebastián Contreras-Riquelme, David S. Holmes and Tomas Perez-Acle

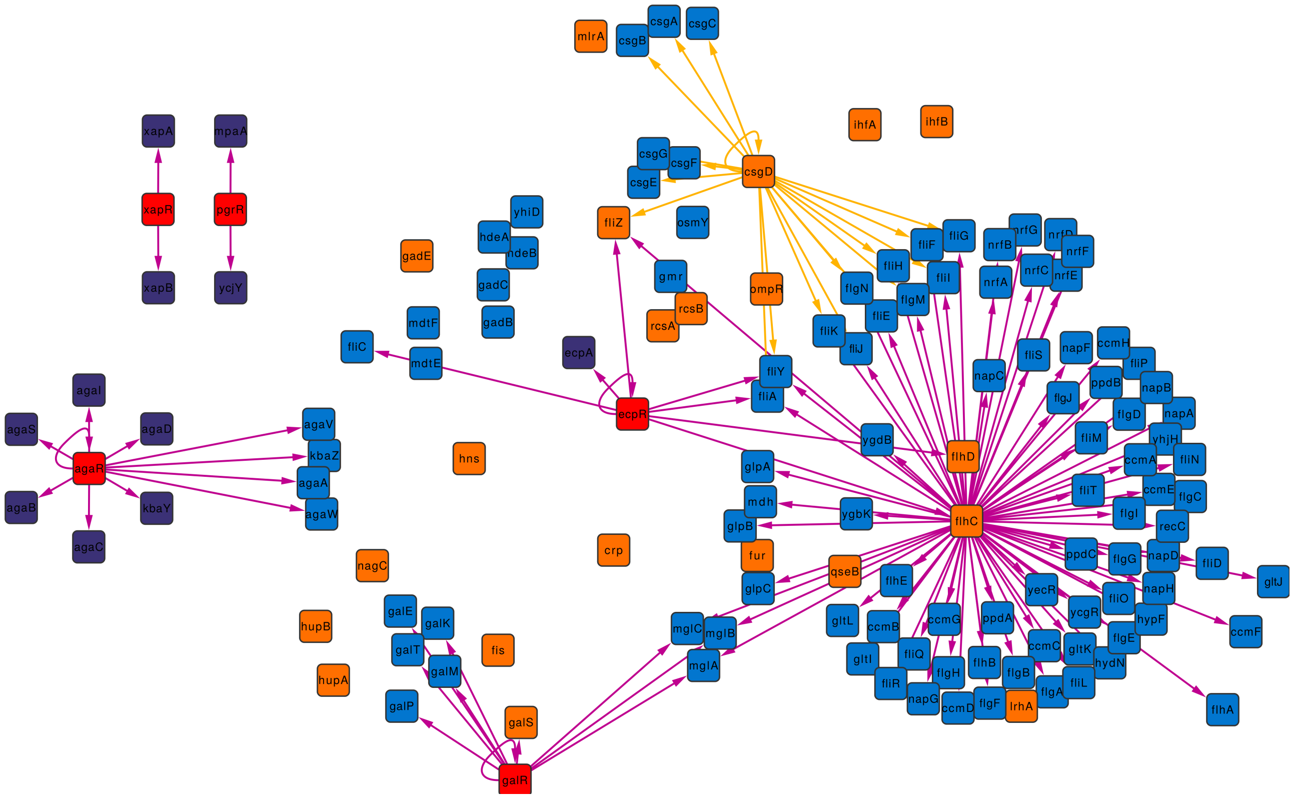


**Merged sub-network of the TFs whose RGD varies the most using Biofilm network at 15 hours as reference. Only FP and FN edges are shown. Color codes are the same as in Figs 4 and 5 in the main text.**
