# Supplementary material for: Graphlet Based Metrics for the Comparison of Gene Regulatory Networks
Source: PLoS One. 2016 Oct 3;11(10):e0163497. doi: 10.1371/journal.pone.0163497 (PMC5047442; doi:10.1371/journal.pone.0163497)
Supplement: S1 Table — This table shows the number of TFs, nodes (genes) and true edges occurring at four time points of the GRN of E. coli cultured in suspension and forming biofilms. (PDF) [file pone.0163497.s006.pdf]

Graphlet Based Metrics for the Comparison of Gene Regulatory Networks:
Table S1: Components of the condition specific and gold standard GRNs

Alberto J.M. Martin, Calixto Dominguez, Sebastián Contreras-Riquelme, David S. Holmes and Tomas Perez-Acle

		**Biofilm**				**Suspension**			
	Gold Standard	4h	7h	15h	24h	4h	7h	15h	24h
TFs	202	157	156	127	168	161	137	115	125
Nodes	1805	1631	1603	1493	1591	1620	1556	1513	1495
True edges	4511	4006	3960	3537	3914	4034	3714	3442	3527

This table shows the number of Transcription Factors (TFs), nodes (genes) and true edges occurring at four time points of the GRN of *E. coli* cultured in suspension and forming biofilms.
