# Supplementary material for: Graphlet Based Metrics for the Comparison of Gene Regulatory Networks
Source: PLoS One. 2016 Oct 3;11(10):e0163497. doi: 10.1371/journal.pone.0163497 (PMC5047442; doi:10.1371/journal.pone.0163497)
Supplement: S2 Table — This table shows the occurrence of each graphlet type and the number of genes that do not form any graphlet (NOG) along four time points when E. coli was cultured in suspension, forming biofilms and in the entire gold standard. Graphlets of types 9 are absent in all these condition specific networks. (PDF) [file pone.0163497.s007.pdf]

Graphlet Based Metrics for the Comparison of Gene Regulatory Networks:
Table S2: Graphlets in condition specific and gold standard GRNs

Alberto J.M. Martin, Calixto Dominguez, Sebastián Contreras-Riquelme, David S. Holmes and Tomas Perez-Acle

		Biofilm				Suspension			
Type	Gold	4h	7h	15h	24h	4h	7h	15h	24h
1	329819	307539	306911	299158	303724	308097	302940	298927	299144
2	6305	5162	4989	4032	4913	5235	4365	3508	4067
3	1634	1505	1504	1477	1573	1505	1328	1263	1506
4	4338	3667	3598	2932	3473	3768	3173	2687	2827
5	1641	1468	1414	1159	1437	1533	1236	1082	1198
6	488	468	470	459	479	468	458	456	460
7	89	73	64	53	79	73	57	52	62
8	5	3	1	1	5	3	1	1	3
9	0	0	0	0	0	0	0	0	0
10	8	6	5	4	7	6	3	4	5
11	31	20	23	18	28	20	17	16	19
12	3	3	3	3	3	3	3	3	3
13	1	1	1	1	1	1	1	1	1
NOG	11	8	6	6	8	8	6	6	4

This table shows the occurrence of each graphlet type and the number of genes that do not form any graphlet (NOG) along four time points when *E. coli* was cultured in suspension, forming biofilms and in the entire gold standard. Graphlets of types 9 are absent in all these condition specific networks.
