# Supplementary material for: Graphlet Based Metrics for the Comparison of Gene Regulatory Networks
Source: PLoS One. 2016 Oct 3;11(10):e0163497. doi: 10.1371/journal.pone.0163497 (PMC5047442; doi:10.1371/journal.pone.0163497)
Supplement: S3 Table — Columns in this table show, from left to right, Spearman’s correlation (ρ); Kendall’s correlation (τ); Pearson correlation (r); The rate of disagreement (d = nodes identified only by one metric/# nodes); The rate of disagreement for nodes with variations in the metric (dc = nodes identified only by one metric/nodes identified only by one or the two metrics metric); The number of nodes identified by both metrics (Both); only by RGD (RGD); only by the other metric (M); and the total number of nodes (#). The comparisons were performed using only those nodes for which both RGD and the centrality measurements could be computed using the Biofilm networks as reference (BS) and the Suspension network as reference (SB). Centrality metrics are (Cent): Average Shortest Path Length (ASPL); Betweenness Centrality (BC); Closeness Centrality (CC); Clustering Coefficient (CLC); Eccentricity (E); Edge Count (EC); Indegree (ID); Outdegree (OD); Neighborhood Connectivity (NC); and Stress Centrality (SC). See below for a definition of the centralities used. This file also contains the definition of the centrality metrics employed and several figures showing the comparison of these centrality metrics versus RGD on the comparisons performed using the biofilm and suspension networks at 15 hours as reference. (PDF) [file pone.0163497.s008.pdf]

Graphlet Based Metrics for the Comparison of Gene Regulatory Networks: Table S3: Comparison of RGD with other centrality metrics in condition specific networks at 15 hours

Alberto J.M. Martin, Calixto Dominguez, Sebastián Contreras-Riquelme, David S. Holmes and Tomas Perez-Acle

Comp.	Cent	ρ	τ	r	d	dc	Both	RGD	M	#
BS	ASPL	-0.265	-0.247	-0.174	0.248	0.898	41	358	3	1456
	BC	-0.217	-0.201	-0.110	0.253	0.898	42	357	12	1456
	CC	-0.265	-0.247	-0.176	0.248	0.898	41	358	3	1456
	CLC	-0.496	-0.455	-0.232	0.190	0.636	158	241	35	1456
	E	-0.216	-0.202	-0.172	0.259	0.945	22	377	0	1456
	EC	-0.643	-0.598	-0.314	0.157	0.516	215	184	45	1456
	ID	-0.629	-0.587	-0.449	0.161	0.527	210	189	45	1456
	OD	-0.186	-0.174	-0.253	0.264	0.965	14	385	0	1456
	NC	-0.604	-0.508	-0.065	0.231	0.520	310	89	247	1456
	SC	-0.228	-0.212	-0.128	0.251	0.899	41	358	8	1456
SB	ASPL	-0.219	-0.201	-0.207	0.325	0.927	37	466	7	1456
	BC	-0.152	-0.138	-0.096	0.334	0.933	35	468	19	1456
	CC	-0.219	-0.200	-0.314	0.325	0.927	37	466	7	1456
	CLC	-0.430	-0.385	-0.246	0.266	0.716	154	349	39	1456
	E	-0.192	-0.176	-0.170	0.333	0.960	20	483	2	1456
	EC	-0.290	-0.264	-0.159	0.318	0.755	150	353	110	1456
	ID	-0.276	-0.252	-0.214	0.320	0.761	146	357	109	1456
	OD	-0.163	-0.149	-0.130	0.337	0.974	13	490	1	1456
	NC	-0.631	-0.563	-0.122	0.159	0.359	414	89	143	1456
	SC	-0.172	-0.157	-0.063	0.331	0.932	35	468	14	1456

Columns show, from left to right, Spearman's correlation (ρ); Kendall's correlation (τ); Pearson correlation (r); The rate of disagreement (d = nodes identified only by one metric/# nodes); The rate of disagreement for nodes with variations in the metric (dc = nodes identified only by one metric/nodes identified only by one or the two metrics metric); The number of nodes identified by both metrics (Both); only by RGD (RGD); only by the other metric (M); and the total number of nodes (#). The comparisons were performed using only those nodes for which both RGD and the centrality measurements could be computed using the Biofilm networks as reference (BS) and the Suspension network as reference (SB). Centrality metrics are (Cent): Average Shortest Path Length (ASPL); Betweenness Centrality (BC); Closeness Centrality (CC); Clustering Coefficient (CLC); Eccentricity (E); Edge Count (EC); Indegree (ID); Outdegree (OD); Neighborhood Connectivity (NC); and Stress Centrality (SC). See below for a definition of the centralities used.

Centrality Metrics: Let $G = (V, E)$ be a graph formed by a set of vertices or nodes (V) and a set of edges (E). Sub-indices i and j represent specific nodes (v_i and v_j respectively) and $A_{i,j}$ values of the adjacency matrix that represent G. Each position in this matrix can be assigned two values, $A_{i,j} = 1$ if the product of node v_i regulates the expression of node v_j, and $A_{i,j} = 1$ if the product of node v_i does not regulates node v_j.

- In-degree (ID or k^-): $k^-(i) = \sum_{j \in V} A_{ji}$;
- Out-degree (OD or k^+): $k^+(i) = \sum_{j \in V} A_{ij}$;
- Edge count or degree (EC or k): $k(i) = \sum_{j \in V}^{n} (A_{ij} + A_{ji}) = k^-(i) + k^+(i)$;
- Clustering Coefficient (CLC): $C(i) = \frac{e_i}{(k_i(k_i-1))}$;
  where e_i is the number of connected neighbors of node i and k_i its degree.
- Neighborhood connectivity (NC): $NC(i) = \frac{\sum_{j \in N(i)} k_j}{k_i}$;
  where $N(i)$ is the set of neighbors of node i and k_n is the degree of node n.
- Average Shortest Path Length (ASPL): $ASPL(i) = avg(L(i,j)), \forall j \neq i$;
  where $L(i,j)$ is the length of the shortest path from node i to node j.
- Betweenness Centrality (BC or Cb): $Cb(i) = \sum_{j \neq i} \sum_{k \neq i,j} (\sigma_{jk}(i)/\sigma_{jk})$;
  where σ_{jk} is the number of shortest paths from node j to node k and $\sigma_{jk}(i)$ is the number of shortest paths from j to k in which node i lies on.
- Closeness Centrality (CC or Cc): $Cc(i) = \frac{1}{avg(L(i,j))} = \frac{1}{ASPL(i)}$;
- Eccentricity (E): $E(i) = max(L(i,j)), \forall j \neq i$;
- Stress Centrality (SC or Cs): $Cs(i) = \sum_{j \neq i} \sum_{k \neq i,j} \sigma_{jk}(i)$;
  where $\sigma_{jk}(i)$ is the number of shortest paths from j to k in which node i lies on.

Comparison of centrality metrics versus RGD on the comparisons performed using the Biofilm network at 15 hours as reference:

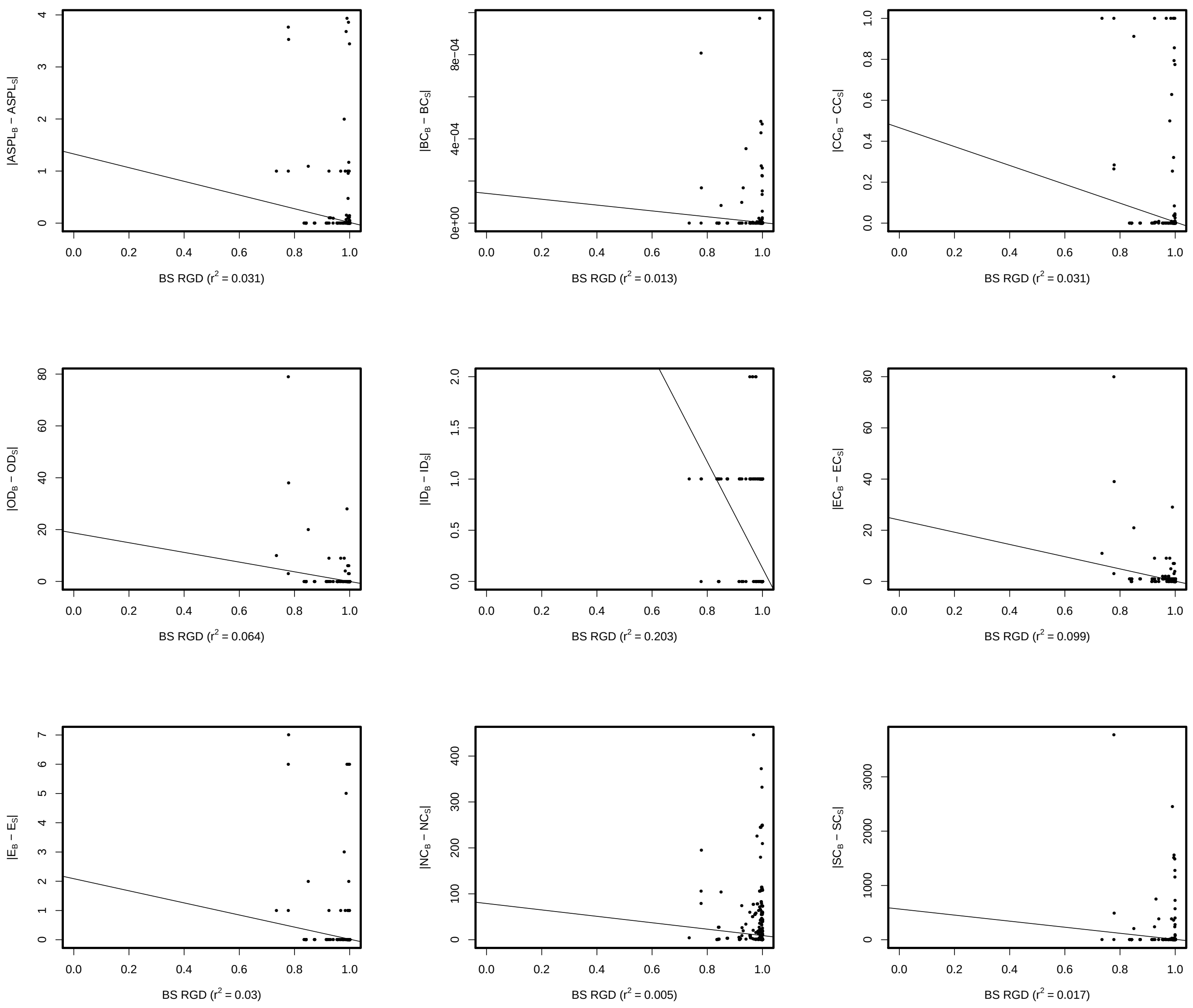

Plots showing the relationships between the differences in centrality metrics and RGD on the same data as in Table S3. Regression lines and regression coefficients (shown between brackets in the x-axis title) were calculated using R using RGD as dependent variable.

Comparison of centrality metrics versus RGD on the comparisons performed using the Suspension network at 15 hours as reference:

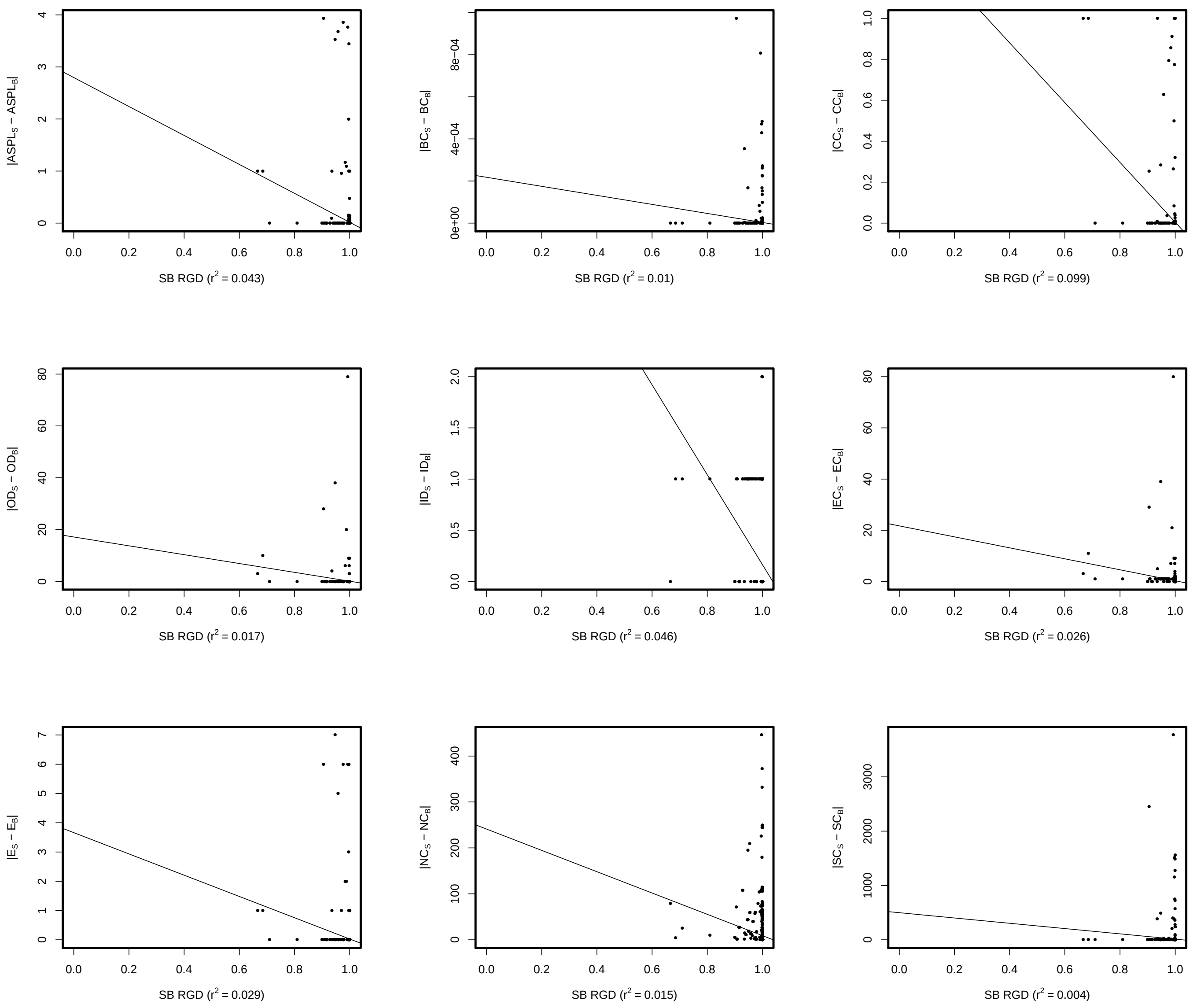

Plots showing the relationships between the differences in centrality metrics and RGD on the same data as in Table S3. Regression lines and regression coefficients (shown between brackets in the x-axis title) were calculated using R using RGD as dependent variable.
