# Supplementary material for: Graphlet Based Metrics for the Comparison of Gene Regulatory Networks
Source: PLoS One. 2016 Oct 3;11(10):e0163497. doi: 10.1371/journal.pone.0163497 (PMC5047442; doi:10.1371/journal.pone.0163497)
Supplement: S1 Text — This text explains the procedure followed to generate random GRNs of any given size and to randomize the E. coli gold standard. The file also shows the performance of the method with respect to network size and the values of REC and RGD on comparisons of randomized E. coli with the reference network. (PDF) [file pone.0163497.s009.pdf]

Graphlet Based Metrics for the Comparison of Gene Regulatory Networks: Text S1: Assessing REC and RGD on comparisons of random networks

Alberto J.M. Martin, Calixto Dominguez, Sebastián Contreras-Riquelme, David S. Holmes and Tomas Perez-Acle

Generation of random GRNs:

Random networks of any given size: Gene Regulatory Networks (GRNs) have some characteristics that make different to other networks. First, edges in them can be originated from a subset of all their nodes, i.e., Transcription Factor (TF) encoding genes. The proportion of origin nodes (n_o) is an order of magnitude smaller than the number of total genes (n) and approximately 10% of the total number of genes. Second, the out-degree distribution follows a power-law distribution, which implies that most of the TF encoding genes are the origin of very few edges and few TF encoding genes have the highest out-degree. Third, GRNs have very low density (ratio between the number of actual edges, m, and all possible edges, n^2 in directed networks), implying that most of the nodes connected to few of the other nodes, i.e., GRNs are sparse. And fourth, the maximum out-degree of a TF encoding gene is smaller than the number of genes in the network (a TF encoding gene does not control the expression of all the genes, just a fraction of them).

To generate random networks that accomplish with these characteristics, the following algorithm was used as an initial step toward the creation of random GRNs with similar characteristics to actual GRN:

Given m; n; n_o; E = {}; $|V| = n$; $V_o \subset V$; and $|V_o| = n_o$;

While $|E|$ < m; do
  select random i $\in V_o$, with $k_i^+ < 2 * (\log(m))^2$;
  select random j $\in V$ given that $\{i, j\} \notin E$;
  if(rand(1) < $\frac{1+k_i^+}{log(1+|E|)^2}$)
    add {i,j} to E;
done;

One should keep in mind that the only purpose of networks created with this algorithm is to test the applicability and scalability of the REC and RGD on networks of various sizes. Initial analysis of these random networks show certain similarities with observed features on real GRNs, but further and detailed analysis is out of the scope of this work.

Randomization of the *E. coli* network: Three different types of simple randomization procedures were applied to the *E. coli* to establish basal values of REC and RGD, their values on comparisons with random networks. Similar to Ruan et al BMC Bioinformatics 2015, the identities of nodes were randomized while maintaining the edges so the topology and properties of the random network remains unaltered. This was done randomly shuffling the ids of both TF encoding genes and non-TF encoding genes, only TF encoding genes and only non-TF encoding genes, denoted RALL, RTF and RNTF respectively. In the RTF networks, each non-TF node maintains their in-degree, but the out-degree, and therefore the graphlets in which each TF participates, change. The opposite happens in RNTF, non-TF change their local topology while TF do not.

In order to establish a basal value of REC and RGD, the *E. coli* gold standard network was randomized in two different ways. First, randomly chosen true connections were removed by transforming them into false edges. This procedure is termed *REMO* hereinafter. Second, randomly selected true connections were transformed into false edges, and for each true edge that was transformed, a randomly selected false edge was transformed into a true edge. Hence, the randomized network maintains the same number of true edges as in the original network. This procedure is termed *SWAP* hereinafter. The two randomization procedures were repeated varying the percentage of changed edges from 0% to 100%. By removing true edges they were transformed into False Negative (FN) edges, i.e., edges only present in the randomized network, in the REMO case. On the other hand, in the SWAP case, removed true links were transformed into FN edges and removed True Negatives (TNs) were transformed into False Positive (FP) edges, i.e.m regulatory connections absent in both networks were transformed to true edges in the randomized GRN. These randomizations were intended to evaluate the behavior of the metrics using a dataset for which the actual percentage of change produced by random alterations is known. To reduce possible dependences on the randomization and to allow proper statistical comparisons, both protocols were repeated 1×10^3 times with a different seed for the random number generator each time.

Performance of the method with respect to network size:

Four different set of parameters were used to generate 100 random networks per set using the algorithm described above. The values of the parameters were chosen to maintain the same proportion as in the *E. coli* reference network described in the main text. The results shown correspond to comparisons performed without writing the network files that are generated in the webserver and without writing the lists of graphlets present in both networks and those that appear only in either network, since here, we are only testing the applicability of the main algorithm used to assign graphlets in the two networks and the comparison of these graphlets.

Size	Avg. TFs	Avg. genes	Avg. time	RAM
100000	1962.51 (5.57)	19879.27 (11.28)	40814.10 (8239.88)	10.1
75000	1471.80 (5.57)	14909.39 (8.46)	19735.56 (3758.16)	7.5
50000	981.28 (4.25)	9940.47 (6.53)	8605.06 (1171.35)	4.9
25000	490.64 (2.72)	4971.82 (5.33)	3365.95 (142.12)	2.4

Table Text S1.1: **Average performance of the method with respect to network size for 100 comparisons between random networks.** Size is the number of true edges (m); Avg. TFs is the average number of TF encoding genes with out-degree ≥ 1; Avg. genes is the average number of genes with in-degree ≥ 1; Avg. time is the averaged time in seconds; and RAM is the maximum RAM memory used by the script in GBs. This test was carried out in a cluster using AMD Opteron(tm) 6376 processors.

Basal values of REC and RGD on comparisons of randomized *E. coli* with the reference network

As can be seen in Fig. Text S1.1 and Fig. Text S1.2 basal values for both REC and RGD are relatively far away from their theoretical minima of 0. Basal values depend on the gene type (TF and non-TF encoding) and on the randomization protocol applied to the reference network.

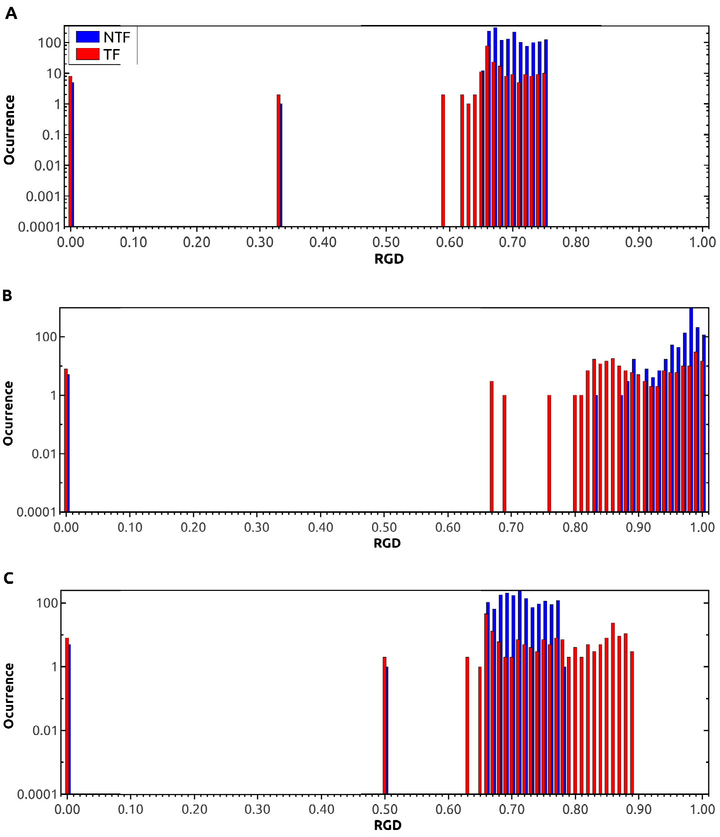

Figure Text S1.1: **Occurrence of different per gene averaged RGD values.** Averaged RGD for each gene on comparisons of the original GRN with 10^3 replica of the three procedures applied to the *E. coli* GRN. Panel A) shuffling of all gene names or RALL; Panel B) shuffling of only TF encoding gene names or RTF; and Panel C) only non-TF encoding genes or RNTF. Blue bars represent non-TF encoding genes, red bars TF encoding genes. Ocurrence (Y-axis) is in log10 scale.

The results for the three different randomizations shown in Fig. Text S1.1 determine the behavior of RGD in three different scenario in which the network structure remains unaltered. In the RALL randomization, both TF and non-TF encoding genes show averaged RGD values no lower than 0.65, with few exceptions for TF-encoding genes. In this case, the RGD minimum is placed far away from its theoretical minima of 0, with

higher values for non-TF-encoding genes than for TF-encoding genes. When only the ids of the TF-encoding genes where randomized (RTF), non-TF-encoding genes have RGD values relatively high (the most frequent is 0.98), while TF-encoding nodes have RGD values more widespread. The last randomization of this type, RNTF, presents RGD values for non-TF-encoding genes that are significantly lower than in the RTF randomization but more similar to those in RALL. In this last case, RGD values for non-TF-encoding genes are more evenly spread over the 0.65 to 0.77 range than in RALL, and TF-encoding genes show more similar values to those in the RTF randomization (even if in this case their RGD values are also more spread).

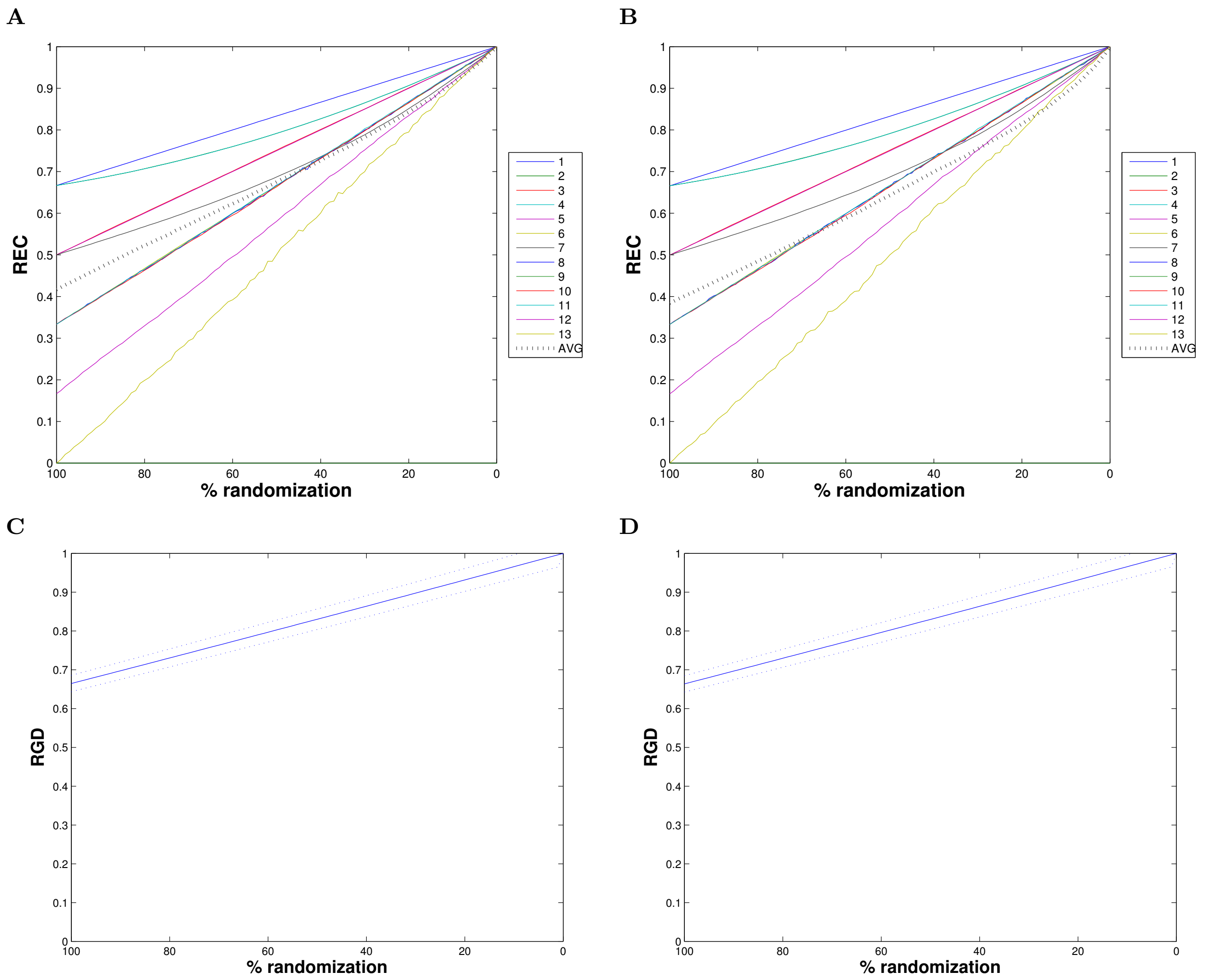

Figure Text S1.2: **Averaged REC and RGD values on the 10^3 replica of REMO and SWAP randomized networks.** Averaged REC for each graphlet types and average for all graphlet types on the REMO and SWAP randomizations (panels A and B respectively). Averaged RGD value for all gene types (TF and non-TF encoding) on the REMO and SWAP randomizations (panels C and D respectively).

The averaged REC for each type of graphlet are very similar in both REMO and SWAP randomization procedures, with slightly lower values for the later (Fig. Text S1.2, panels A and B). As for RGD in the RALL, RTF and RNTF randomizations, averaged REC for each graphlet type is also far away from its theoretical minima of 0, with the only exception of the only graphlet of type 13 present in the *E. coli* reference network. Interestingly, types 1 and 4, the most common graphlet types also have the highest averaged REC, while the less frequent graphlets that require their three nodes to depict TF-encoding genes (types 7 to 13) have the lowest REC values. Regarding averaged RGD values on the same randomizations (Fig. Text S1.2, panels C and D), these decrease linearly as the percentage of random edges in the networks increase, reaching an approximate minima of 0.67 in both cases. The relatively reduced standard deviations of the averaged RGD (doted lines), are an indicator of the metric robustness since the averaged RGD shown belong to 10^3 replica of the randomizations started each of them with a different seed for the random number generator. With respect to the averaged values for TF and non-TF encoding genes (not shown for clarity), these are very similar to the global average with TF-encoding values slightly below.

Both REC and RGD values, as mentioned above, are far from their theoretical minima of 0 in all randomizations. This indicates that both metrics are dominated by the false edges (non-existing edges or non-regulations) that due to their prevalence remain unaltered. This happens independently of how the GRN was randomized, thus, confirming the trend for all five randomization approaches. Nevertheless, RGD is still able to identify those nodes whose local topology varies in the comparison of two states of a GRN and the averaged REC for all graphlet types is a good indicator of the topological similarity between different versions of a GRN.
