# Supplementary material for: Graphlet Based Metrics for the Comparison of Gene Regulatory Networks
Source: PLoS One. 2016 Oct 3;11(10):e0163497. doi: 10.1371/journal.pone.0163497 (PMC5047442; doi:10.1371/journal.pone.0163497)
Supplement: S2 Text — (PDF) [file pone.0163497.s010.pdf]

Graphlet Based Metrics for the Comparison of Gene Regulatory Networks: Text S2: Functional characterization of genes coding TFs with lowest RGD at 15 hours

Alberto J.M. Martin, Calixto Dominguez, Sebastián Contreras-Riquelme, David S. Holmes and Tomas Perez-Acle

This analysis was performed with information obtained from RegulonDB [1] and EcoCyc [2].

Genes coding for TF with the lowest RGD using biofilm as reference:

pgrR is a repressor of the initial enzymes for petidoglycan peptide (aka mureine, forms cell wall) degradation and genes of the switch control between peptidoglycan recycling and degradation. pgrR forms a single graphlet of type 1 that is only present in the biofilm subnetwork, since its expression was only detected in the biofilm experiments. A value of 0.667 for the REC of this single graphlet means that the four false edges existing among the three nodes are the only ones that are maintained in the suspension network.

xapR activates transcription of genes involved in xanthosine metabolism when xanthosine and deoxyinosine are present. xapR forms a single graphlet of type one in the biofilm network that was not found in the suspension network, this was caused by the absence of xapR expression in the suspension condition. Its REC value of 0.667 means, as happened with pgrR, that only the four false edges existing in the biofilm network between the three nodes participating in the single type 1 graphlet are maintained.

agaR is a negative regulator of its own gene and of other genes involved in transport and catabolism of N-acetylgalactosamine and d-galactosamine (kbaZ-agaVWA and agaS-kbaY-agaBCDI operons). The expression of agaR was only detected in biofilm, while it was absent in suspension. The expression of kbaZ-agaVWA operon is induced by crp, a Transcription Factor (TF) whose expression is detected in both conditions. Formation of DHAP (Dihydroxyacetone phosphate) from galactosamine and galactitiol repress AI-2 uptake in early biofilm formation (4 and 7 hours) [3]. AI-2 increases biofilm formation as its uptake increases [3]. The products of genes agaB and agaC are the enzymes directly involved in the formation of DHAP from galactosamine, their deletion increases biofilm formation [3].

galR represses transcription of itself and of the operons whose products are involved in transport and catabolism of D-galactose. Galactose is a precursor of galactosamine. The subnetwork of galR shares several TFs with the subnetwork of ecpR, a major controller of flagellum biosynthesis. The mglBAC operon is regulated by the products of flhD and flhC, both regulated by ecpR. crp (also part of the agaR subnetwork), one of the nodes with higher participation in graphlets and also present in the subnetwork of ecpR, induces the expression of the mglBAC operon

ecpR is a positive regulator acting at transcriptional and posttranscriptional levels, in the expression of the mat operon; it also controls the expression of the flhDC operon, repressing flagellum biosynthesis, motility, and taxis. The expression of ecpR is only detected in biofilm, while in suspension is absent. ecpR is a fimbriae activator, it switches off motility in *E. coli* by repression of the flagellar master operon flhDC (genes flhC and flhD). The opposite regulatory actions of ecpR on mat and on flhDC induce the adaptation of *E. coli* from a planktonic to an adhesive lifestyle. ecpR binds weakly to pfliA and to pfliC, and their levels are equal to those observed for matB. ecpR is tightly integrated into two separate regulatory circuits, hns-ecpR-flhDC and rcsB-ecpR-flhDC, both of which control expression of the flagellum. Flagellar genes are induced in all stages during biofilm formation [3].

Table S1 Text.1: **Participation in each graphlet type of the TFs with the highest decay in RGD using biofilm as reference.**

TF	1	2	3	4	5	6	7	8	9	10	11	12	13	total
pgrR	1	0	0	0	0	0	0	0	0	0	0	0	0	1
xapR	1	0	0	0	0	0	0	0	0	0	0	0	0	1
agaR	45	0	0	4	0	0	0	0	0	0	0	0	0	49
galR	28	0	0	29	0	8	1	0	0	0	0	0	0	66
ecpR	15	170	0	25	4	0	0	0	0	0	2	0	0	216

Genes coding for TFs with the lowest RGD using suspension as reference:

dicA is a temperature-sensitive repressor that controls the transcription of genes involved in the cell division process and activation of its own expression. Its expression is solely detected in suspension, even thought the signal to which dicA responds is still unknown, and therefore all graphlets in which it participates (21 type 1 graphlets) are absent in biofilm. Genes regulated by dicA are related to prophage and phage related functions. Its average REC of 0.667 indicates that the four false edges of each of the 21 type 1 graphlets in which it participates, are the only edges maintained in the biofilm network.

nsrR regulates negatively genes involved in cell protection against nitric oxide (NO). Its expression was only detected in the suspension network. The operon containing the nsrR gene is induced by cold shock. nsrR inhibits the expression of more than 30 genes, several involved in aerobic respiration and in fermentation. The subnetwork of nsrR also includes several nodes that are also present in the subnetwork of ecpR, indicating a relationship between these two TFs.

norR is one of several regulatory proteins, such as Fur, SoxR, and OxyR, that are involved in the response to reactive nitrogen species. Under anaerobic and micro-aerobic conditions it activates transcription of the norVW operon, encoding a nitric oxide (NO)-reducing flavorubredoxin that detoxifies NO. The norVW operon is also regulated by ihfA-ihfB (+/-) and negatively by narP, narL and fnr. The two genes in the norVW operon, norV and norW, are not expressed in the two different networks. norR expression was detected in both conditions, it inhibits its own expression and it is also negatively regulated by nsrR.

paaX regulates negatively the expression of genes involved in the catabolism of an aromatic compound, phenylacetic acid. Phenylacetate is a source of carbon and energy. paaX was only expressed in the suspension network.

mntR is a dual regulator that senses manganese and negatively or positively controls the transcription of different genes involved in manganese homeostasis. mntR co-regulates negatively with fis and hns, dps. the expression of dps is induced by ihfA-ihfB, both present in the two conditions, and oxyR (oxyR is absent in all 8 experiments). dps is a stationary phase nucleoid component that sequesters iron and protects DNA from damage, highly abundant protein in stationary-phase of E. coli and it is required for the normal starvation response and long-term stationary phase viability. The product of dps is involved in protection from multiple stresses, including oxidative stress, the stress of exposure to visible light in a seawater environment or under conditions of fatty acid starvation, and N-ethylmaleimide treatment.

Table S1 Text.2: **Participation in each graphlet type of the TFs with the highest decay in RGD using suspension as reference.**

TF	1	2	3	4	5	6	7	8	9	10	11	12	13	total
dicA	21	0	0	0	0	0	0	0	0	0	0	0	0	21
nsrR	3210	291	0	128	30	0	3	0	0	0	0	0	0	3662
norR	81	2	0	10	0	0	0	0	0	0	0	0	0	93
paaX	78	0	0	36	0	0	0	0	0	0	0	0	0	114
mntR	3	0	0	6	0	0	0	0	0	0	0	0	0	9

References

[1] Salgado H, Peralta-Gil M, Gama-Castro S, et al. RegulonDB v8.0: omics data sets, evolutionary conservation, regulatory phrases, cross-validated gold standards and more. Proceedings of the National Academy of Sciences. 2013 Jan;41(Database issue):D203–13.

[2] Keseler IM, Mackie A, Peralta-Gil M, et al. EcoCyc: fusing model organism databases with systems biology. Nucleic Acids Research. 2013 Jan;41(Database issue):D605–12.

[3] Domka J, Lee J, Bansal T, Wood TK. Temporal gene-expression in Escherichia coli K-12 biofilms. Environmental Microbiology. 2007 Feb;9(2):332–46.
